# Visfatin levels in pulmonary disease: a systematic review and meta-analysis

**DOI:** 10.3389/fmed.2025.1541595

**Published:** 2025-09-19

**Authors:** Muhammad Islampanah, Reza Hossein Zadeh, Abolfazl Akbari, Hamed Ghoshouni, Mehrnush Saghab Torbati, Javad Ghasemi, Raheleh Ganjali, Masoumeh Sadeghi, Mahnaz Mozdourian

**Affiliations:** ^1^Faculty of Medicine, Mashhad University of Medical Sciences, Mashhad, Iran; ^2^Cardiovascular Epidemiology Research Center, Rajaie Cardiovascular Institute, Tehran, Iran; ^3^Department of Medicine, Islamic Azad University, Zahedan, Iran; ^4^Department of Medical Informatics, Faculty of Medicine, Mashhad University of Medical Sciences, Mashhad, Iran; ^5^Metabolic Syndrome Research Center, Mashhad University of Medical Sciences, Mashhad, Iran; ^6^Department of Epidemiology, School of Health, Mashhad University of Medical Sciences, Mashhad, Iran; ^7^Lung Diseases Research Center, Mashhad University of Medical Sciences, Mashhad, Iran

**Keywords:** visfatin, chronic obstructive pulmonary disease, asthma, pneumonia, pulmonary disease

## Abstract

**Background:**

Visfatin has been demonstrated to have pro-inflammatory effects and is involved in several respiratory disorders, including chronic obstructive pulmonary disease (COPD), asthma, and pneumonia. However, there are some inconsistent findings. This study aimed to assess the association between serum visfatin levels and COPD, pneumonia, asthma, interstitial lung disease (ILD), and bronchiectasis.

**Methods:**

A systematic review and meta-analysis were conducted following the Meta-analysis of Observational Studies in Epidemiology (MOOSE) guidelines. PubMed, Scopus, and Web of Science databases were searched. Studies including a healthy control group and measuring serum visfatin in patients with COPD, asthma, pneumonia, bronchiectasis, or ILD were included. Stata 17 was used for data analysis.

**Results:**

Fourteen studies were included. None of them were on bronchiectasis. The analysis showed no significant difference between the COPD group and healthy controls in terms of serum visfatin levels (effect size = −0.02, %95CI: [−0.74, 0.69], *p* = 0.95). Similarly, analysis of visfatin levels in asthma studies showed no significant difference between patients and healthy controls (effect size = −1.51, %95CI: [−6.82, 3.79], *p* = 0.58). However, Serum visfatin levels were significantly higher in pneumonia patients compared to healthy controls (effect size = 1.93, %95CI: [0.91, 2.95], *p* < 0.01).

**Conclusion:**

Circulating levels of visfatin may be associated with pneumonia, but not COPD or asthma. However, there are still few studies on the levels of visfatin in COPD, asthma, and pneumonia patients, and there is a need for further investigation.

**Systematic Review Registration:**

PROSPERO, identifier (CRD42023441144).

## 1 Introduction

, Adipokines, also known as adipocytokines, are protein mediators secreted by adipose tissue that play pivotal roles in regulating metabolism and modulating inflammatory responses (1, 2). Among them, visfatin, also known as nicotinamide phosphoribosyl transferase, was initially identified as a pre-B-cell colony-enhancing factor, which promotes the development of early B-cell progenitors in bone marrow (3).

Visfatin is principally recognized by its metabolic regulatory functions. However, it also serves as a potent pro-inflammatory mediator. It stimulates the production of inflammatory cytokines like IL-1β, IL-6, and TNF-*α* in CD14 + monocytes and enhances the surface expression of co-stimulatory molecules (4). Elevated visfatin levels have been observed in various inflammatory conditions, including inflammatory bowel disease, rheumatoid arthritis, and sepsis (4–6). In recent years, a growing body of evidence indicates that visfatin may play a role in respiratory disorders, such as chronic obstructive pulmonary disease (COPD), asthma, pneumonia, and idiopathic pulmonary fibrosis – a type of interstitial lung disease (ILD) (7–14).

Several studies suggest that visfatin is a novel pro-inflammatory factor in COPD (8, 15), although a previous meta-analysis reported a non-significant reduction in visfatin levels among COPD patients. Nonetheless, a significant association was found between visfatin and inflammatory markers like IL-6 and TNF-*α*. (16). Asthma, another chronic inflammatory disease of the airways, shares similar inflammatory mechanisms with COPD. An inverse relationship between plasma visfatin levels and lung function, specifically forced expiratory volume (FEV1), has been observed in female asthma patients, suggesting a role for visfatin in asthma pathogenesis (7, 17). In addition to its role in COPD and asthma, visfatin has been linked to other pulmonary conditions. Higher plasma visfatin levels have been associated with increased markers of systemic inflammation in community-acquired pneumonia, indicating its potential as a prognostic marker in these patients (18). Furthermore, elevated visfatin levels have been detected in idiopathic pulmonary fibrosis (10, 19). Also, visfatin expression has been demonstrated to contribute to inflammation and apoptosis in acute lung injury and viral infections such as H1N1 (12). Moreover, Nigro et al. found that in patients with COPD and bronchiectasis, a chronic inflammatory airway condition, patients had higher adiponectin levels than COPD patients without bronchiectasis (20, 21). However, some inconsistencies have been found in the studies (8, 22, 23).

Considering the emerging evidence of visfatin’s role in inflammation and its potential role in the pathogenesis of these pulmonary diseases, this systematic review and meta-analysis aimed to assess the association between serum visfatin levels and respiratory conditions such as asthma, COPD, pneumonia, bronchiectasis, and ILD.

## 2 Materials and methods

### 2.1 Search and selection

The systematic review and meta-analysis were performed following the Meta-analysis of Observational Studies in Epidemiology (MOOSE) guidelines (24). The study was registered in the “International Prospective Register of Systematic Reviews” (PROSPERO) in 2023 (CRD42023441144). One reviewer (M.I.) identified studies by searching PubMed, Scopus, and Web of Science through June 29, 2023, using the following search terms and their synonyms: “visfatin” AND (“asthma” OR “chronic obstructive pulmonary disease” OR “bronchiectasis” OR “pneumonia” OR “interstitial lung disease”). The detailed search strategies and other synonyms used are outlined in Supplementary Table S1. The initial search strategy was developed using PubMed and its Medical Subject Headings (MeSH) and then applied uniformly across all searched databases. The search was conducted without time restrictions, and only papers in English were included. Furthermore, the reference lists of the included papers, as well as previous reviews and citations from the most recent included studies, were manually reviewed to identify potential additional studies.

### 2.2 Inclusion and exclusion criteria

All included studies were required to satisfy the following criteria to be included: (1) patients had COPD, pneumonia, asthma, ILD, or bronchiectasis, (2) serum levels of visfatin were assessed in both cases and controls, (3) included a healthy control group, and (4) both cases and controls were human.

Exclusion criteria included (1) being *in vitro*, (2) not using human participants, and (3) full-text not available in English.

### 2.3 Eligibility and quality assessment

Two reviewers (M.S. and H.G.) participated in selecting studies based on their titles, abstracts, and full texts. If consensus could not be reached, a third reviewer (M.I.) made the final decision on inclusion. Quality assessment of included studies was independently conducted by two reviewers (M.I. and R.H.) using the Newcastle-Ottawa Scale (NOS) Quality Assessment Scale (Supplementary Table S2) (25). A score ≥ 7 indicated low risk of bias, scores of 5–6 indicated moderate risk, and a score ≤ 4 indicated high risk of bias. Any disagreements were resolved through discussion among the investigators.

### 2.4 Data extraction

Two independent reviewers (M.I. and R.H.) extracted the following data: first author, year of study publication, study design, country, type of disease, disease severity (either stated qualitatively (e.g., mild, moderate, severe) or using approved classifications (e.g., GOLD for COPD)), method of evaluation of visfatin, number of participants, number of each gender, comorbidities, age, body mass index (BMI), and levels of visfatin. Microsoft Excel spreadsheet (Redmond, WA) was used to store the data.

### 2.5 Statistical analysis

The data were presented as mean ± standard deviation (SD). We used Hedges’ g to calculate the standardized mean differences across studies, and 95% confidence intervals (CIs) indicated the estimated effects. A significance level of *p* < 0.05 was applied to all analyses. Heterogeneity was presented using the *I*^2^ and τ2 indices. Based on heterogeneity, the random- or fixed-effects model was used. Publication bias was examined using funnel plots and the Egger’s test (26). Sensitivity analyses were performed using the leave-one-out method. Subgroup analyses were performed for each lung disease, study methodologies, gender, overweight status, and risk of bias based on NOS. Statistical analyses were conducted using Stata 17 software (StataCorp, College Station, TX).

## 3 Results

### 3.1 Studies characteristics

**Figure 1:**
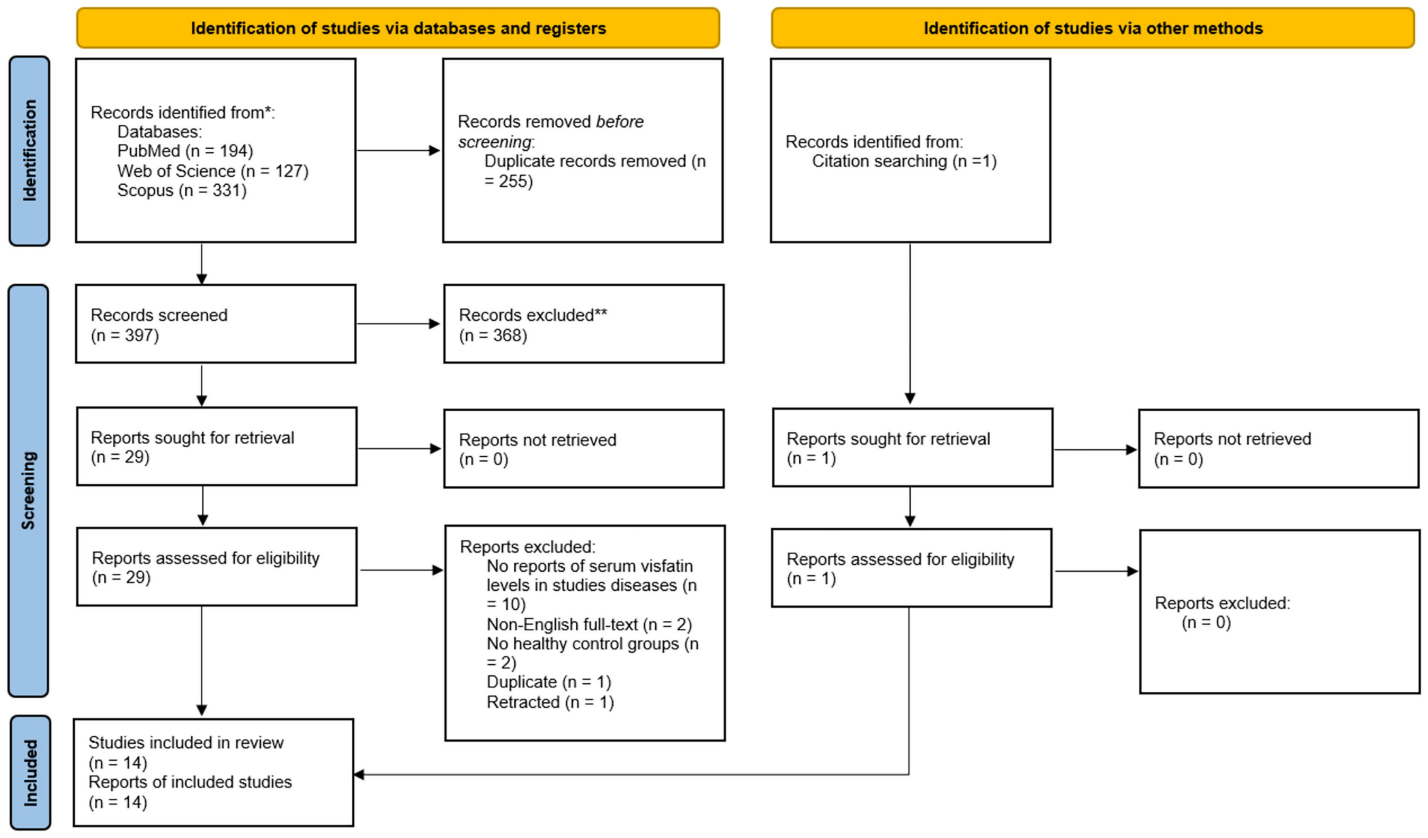
PRISMA flowchart of included studies.

The initial database search identified 397 published studies. After screening the full texts, 14 studies met the inclusion criteria and were included (8, 10, 15, 17, 18, 22, 23, 27–33). None of them were on bronchiectasis, 8 were on COPD, 3 were on asthma, 2 were on pneumonia, and one was on ILD. The detailed process of study selection is shown in Figure 1. Of 14 included studies, five were from Türkiye (17, 22, 27, 29, 33), three from China (15, 18, 31), Italy had 2 (10, 32), and Iran (30), Finland (8), Poland (23), and Mexico (28) each had 1 study (Table 1). Most studies were conducted in Europe (*n* = 9) (8, 10, 17, 22, 23, 27, 29, 32, 33). One was carried out in North America (28), and four were carried out in Asia (15, 18, 30, 31). One study was not included in our meta-analysis since it did not report the numerical value of serum visfatin levels (10). Of the 13 studies included in our meta-analysis, the patient group included 803 cases, and the control group included 458 people.

**Table 1:** Characteristics of included studies.

First Author (year of publication)	Study methodology	Country	Type of disease	Methods of evaluation of visfatin	Severity of disease	# of participants	Comorbidities
COPD							
Leivo-Korpela (2014) (8)	Cross-sectional	Finland	COPD	Enzyme immunoassay	N/A	43 COPD, 41 controls	23% HTN, 12% hypercholesterolemia
Cambay (2021) (22)	Case–control	Türkiye	COPD	ELISA	N/A	37 COPD, 20 controls	N/A
Liu (2009) (15)	Cross-sectional	China	COPD	ELISA	N/A	35 COPD, 28 controls	N/A
Eker (2010) (27)	Cross-sectional	Türkiye	COPD	ELISA	1 mild (excluded from the analysis), 25 moderate, 19 severe, 11 very severe	55 COPD, 25 controls	N/A
Pérez-Bautista (2018) (28)	Cross-sectional	Mexico	COPD	Immunoassay	12 GOLD I, 68 GOLD II, 39 GOLD III, 21 GOLD IV	140 COPD, 70 controls	N/A
Göktepe (2020) (29)	Cross-sectional	Türkiye	COPD	ELISA	mostly moderate-to-severe, 20 GOLD IV	30 COPD, 30 Controls	N/A
Ghobadi (2021) (30)	Case–control	Iran	COPD	ELISA	14 GOLD I-II, 16 GOLD III-IV	30 COPD, 30 controls	N/A
Ayada (2015) (33)	Cross-sectional	Türkiye	COPD	ELISA	N/A	27 COPD, 16 controls	N/A
Asthma							
Machura (2012) (23)	Cross-sectional	Poland	Asthma	ELISA	12 intermittent, 56 mild, 32 moderate persistent	89 asthma, 33 controls	33.7% obesity
Toru (2015) (17)	Cross-sectional	Türkiye	Asthma	ELISA	N/A	27 asthma, 23 controls	N/A
Magrone (2014) (32)	Cross-sectional	Italy	Asthma	N/A	N/A	44 asthma, 17 controls	N/A
Pneumonia							
Juan (2011) (31)	Cross-sectional	China	Pneumonia	ELISA	40 Severe, 30 non-severe	70 pneumonia, 30 controls	N/A
Hu (2013) (18)	Case–control	China	Pneumonia	enzyme immunoassay	PSI score 90.1 ± 22.9, APACHE II score 17.2 ± 7.4	176 pneumonia, 95 controls	4.0% heart failure, 10.2% renal failure, 6.8% liver failure, 21.6% COPD, 13.1% neoplasm, 14.8% neurologic disease, 22.2% HTN, 19.9% DM
Vantaggiato (2023) (10)	Observational (cross-sectional)	Italy	IPF	Western Blot	N/A	23 ILD, 15 controls	N/A

First author (year of publication)	# of males (patients)	# of males (controls)	Male to all participants ratio	Age of patients (years) (mean ± SD)	Age of controls (years) (mean ±SD)	Mean age of all participants	BMI of patients (mean ± SD)	BMI of controls (mean ± SD)	Mean BMI of all participants	Visfatin of patients (ng/mL) (mean ± SD)	Visfatin of controls (ng/mL) (mean ± SD)
COPD											
Leivo-Korpela (2014) (8)	43	41	1	59.5 ± 7.8	N/A	NA	25.8 ± 4.2	26.7 ± 3.9	26.24	7.5 ± 1.5	8.9 ± 2.3
Cambay (2021) (22)	37	20	1	61.59 ± 5.91	60.05 ± 5.71	61.05	21.38 ± 2.86	23.67 ± 1.98	22.18	8.37 ± 1.26	9.71 ± 1.36
Liu (2009) (15)	35	28	1	70 ± 7	70 ± 7	70	18.4 ± 2.3	22.3 ± 3.5	20.13	2.07 ± 0.18	1.88 ± 0.15
Eker (2010) (27)	52	22	0.93	64.47 ± 7.9	63.84 ± 6.2	64.27	24.034 ± 4.2	24.8 ± 2.01	24.27	1.7 ± 1.47	3.27 ± 2.11
Pérez-Bautista (2018) (28)	0	0	0	69.98 ± 8.51	67.26 ± 7.32	69.07	27.01 ± 5.74	26 ± 6	26.67	0.58 ± 0.7	0.71 ± 0.38
Göktepe (2020) (29)	N/A	N/A	NA	64.25 ± 8.58	47.5 ± 13.72	55.88	25.61 ± 6.16	27.55 ± 4.16	26.58	4.66 ± 6.22	8.03 ± 6.64
Ghobadi (2021) (30)	30	30	1	58.83 ± 9.47	56.27 ± 8.12	57.55	26.06 ± 5.3	26.9 ± 3.91	26.48	3 ± 1.56	2.67 ± 0.78
Ayada (2015) (33)	N/A	N/A	NA	N/A	N/A	NA	N/A	N/A	NA	0.00008 ± 0.00001	0.00006 ± 0.00001
Asthma											
Machura (2012) (23)	61	20	0.66	11.34 ± 0.37	11.71 ± 3.79	11.44	20.42 ± 3.13	18.3 ± 0.8	19.85	2.08 ± 0.07	2.57 ± 0.07
Toru (2015) (17)	4	4	0.16	39.3 ± 14.03	45 ± 20.14	42.44	28.3 ± 6.03	24.9 ± 5.87	26.43	0.000093 ± 0.00005	0.000046 ± 0.00003
Magrone (2014) (32)	30	9	0.64	N/A	N/A	NA	N/A	N/A	NA	0.020526 ± N/A	0.00947 ± N/A
Pneumonia											
Juan (2011) (31)	46	19	0.65	51.73 ± 9.98	51.16 ± 11.17	51.56	23.97 ± 4.16	25.31 ± 4.9	24.37	7.03 ± 4.6	1.6 ± 0.26
Hu (2013) (18)	82	N/A	NA	70.1 ± 11.7	N/A	NA	22.4 ± 3.1	N/A	NA	51.6 ± 19.1	13.6 ± 3.5
ILD											
Vantaggiato (2023) (10)	17	5	0.58	75 ± 6.1	54.3 ± 12.9	66.83	N/A	N/A	NA	N/A	N/A

Eight studies were on COPD, three were on asthma, and two were on pneumonia. COPD: chronic obstructive pulmonary disease; ELISA: enzyme-linked immunoassay; GOLD: Global Initiative for Obstructive Lung Disease; DM: diabetes mellitus; HTNL hypertension; IPF: idiopathic pulmonary fibrosis; ILD: interstitial lung disease.

### 3.2 COPD

**Figure 2:**
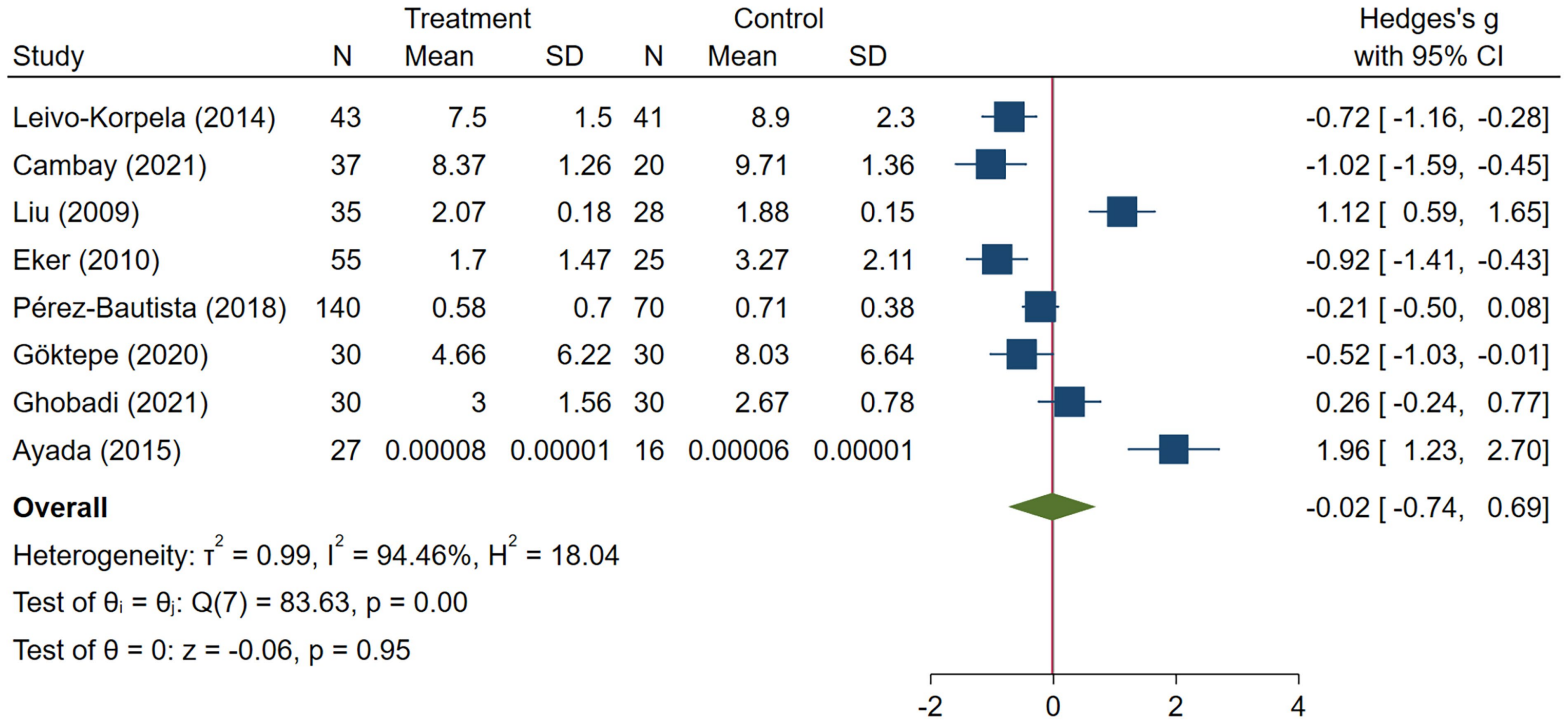
Random effects meta-analysis of COPD studies.

**Figure 3:**
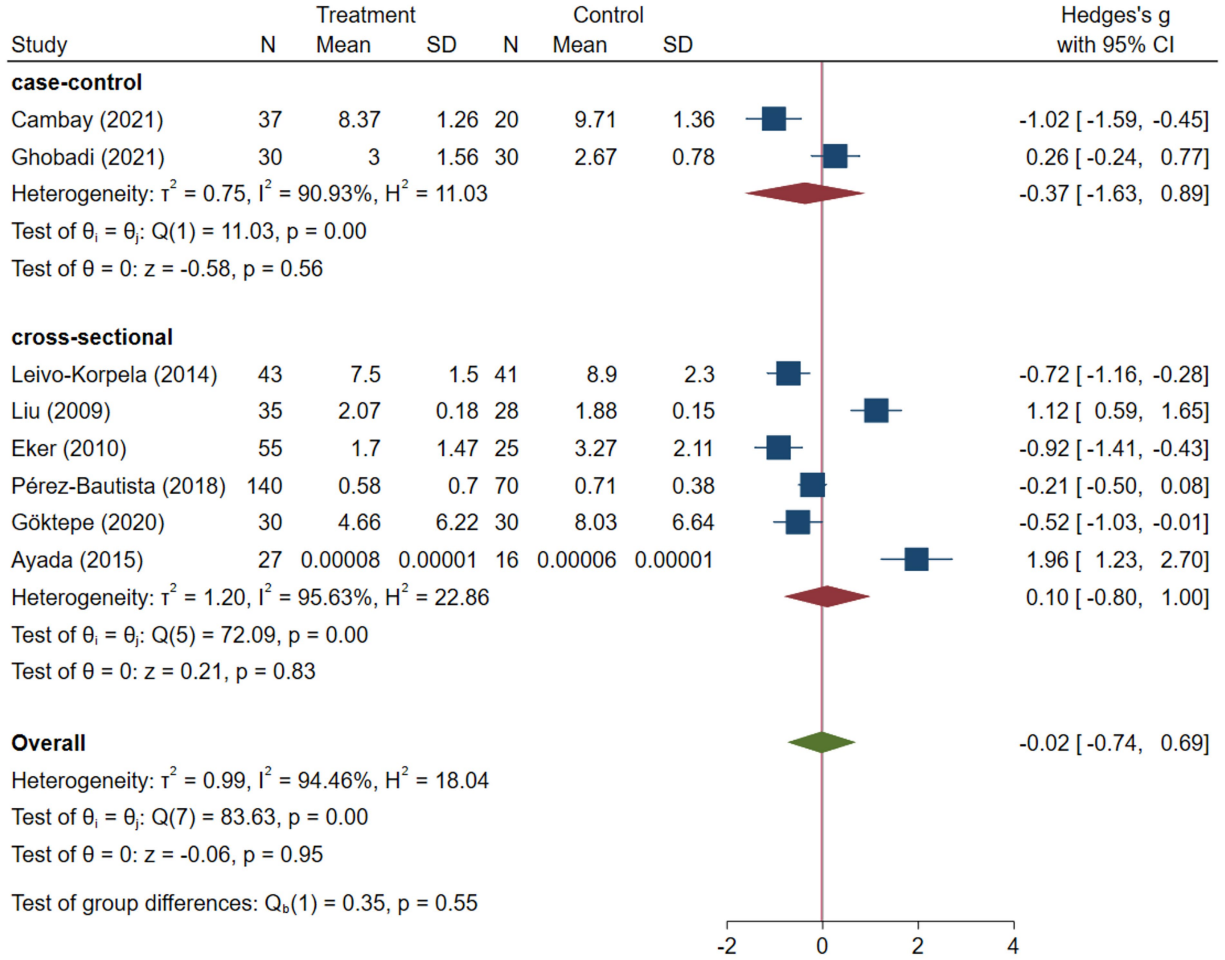
Subgroup meta-analysis of COPD studies based on study methodology.

**Figure 4:**
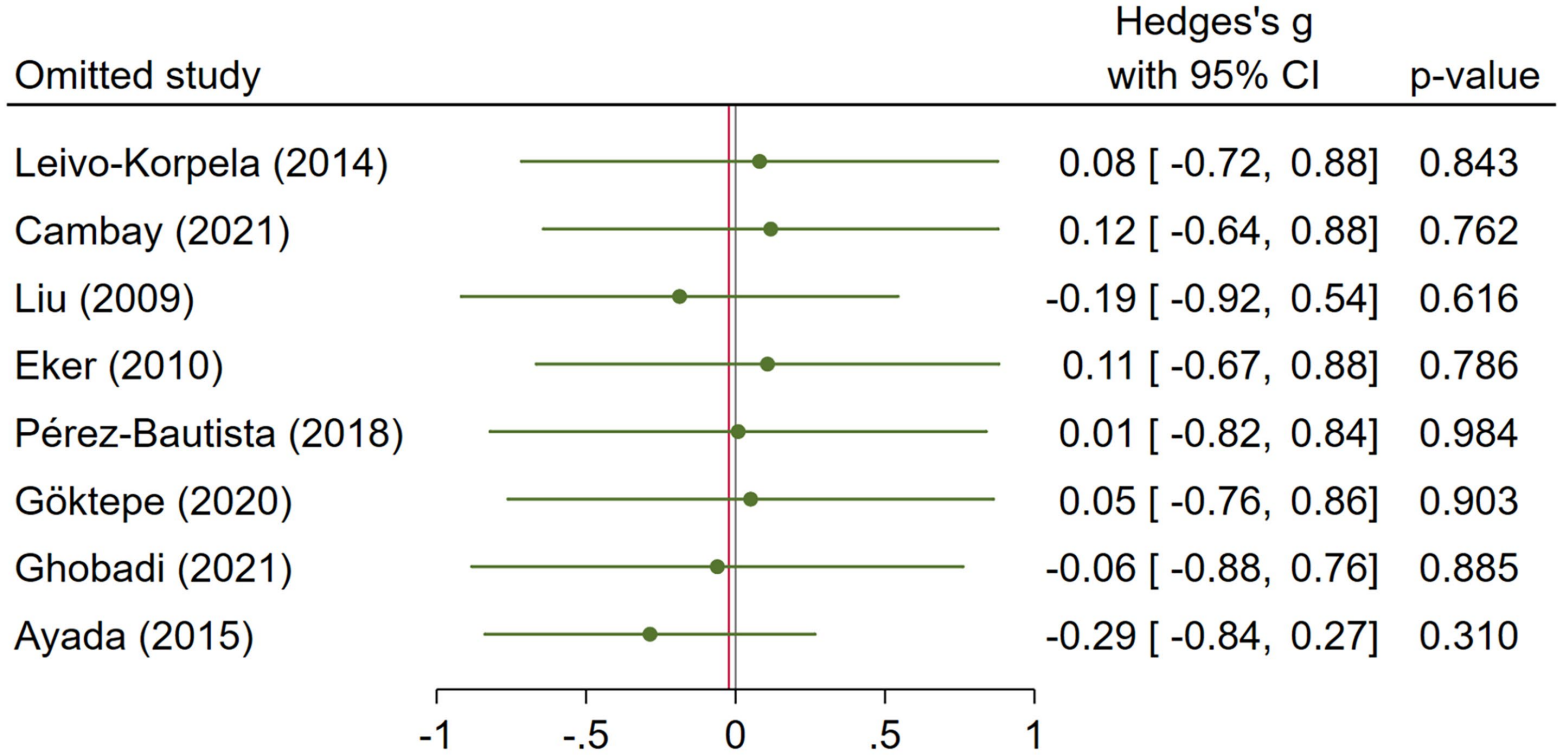
Leave-one-out sensitivity analysis of COPD studies.

**Figure 5:**
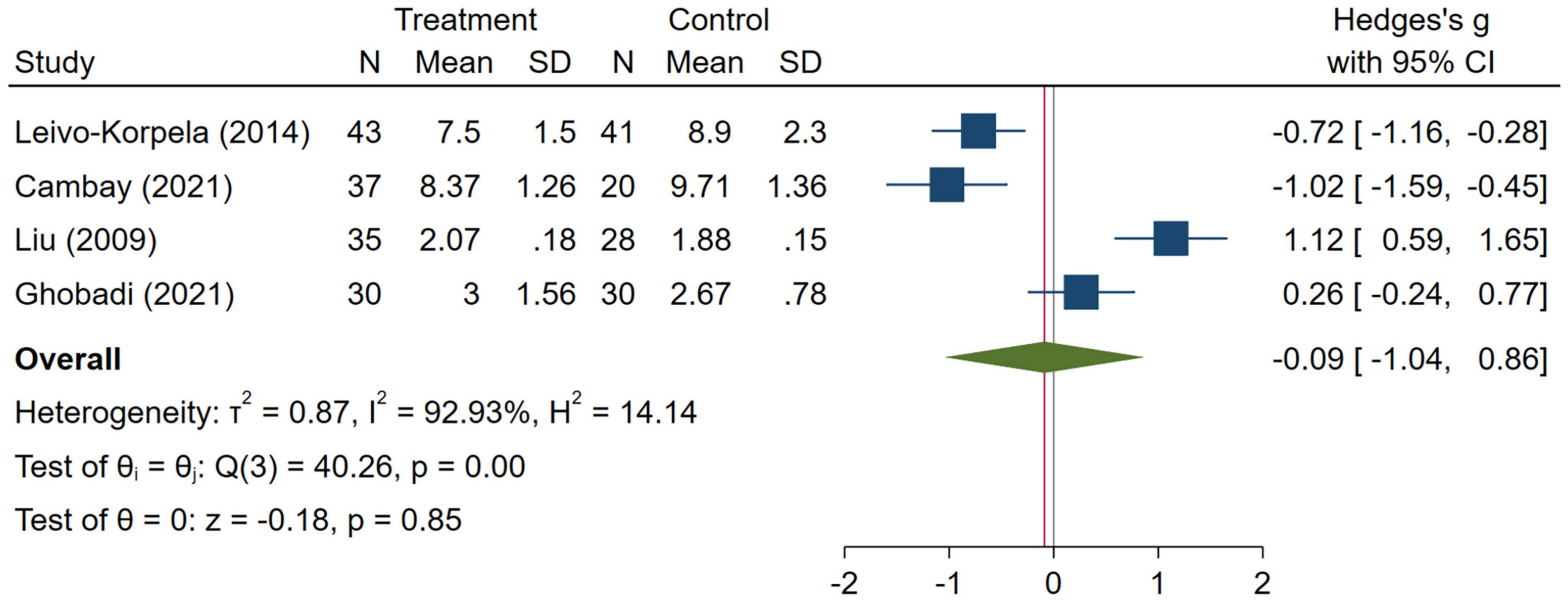
Subgroup analysis on COPD studies which involve only male participants.

**Figure 6:**
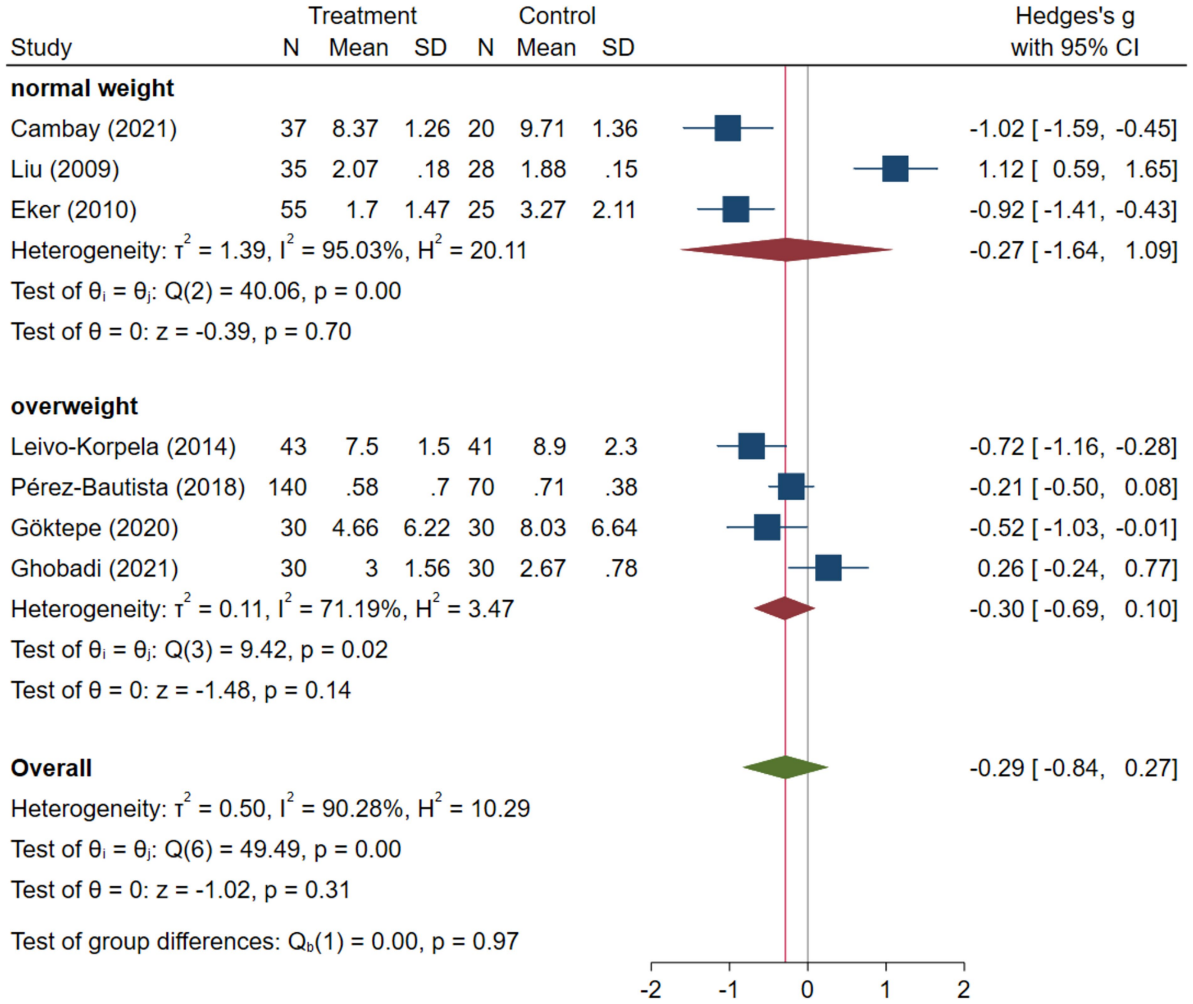
Subgroup analysis on COPD studies based on weight status.

**Figure 7:**
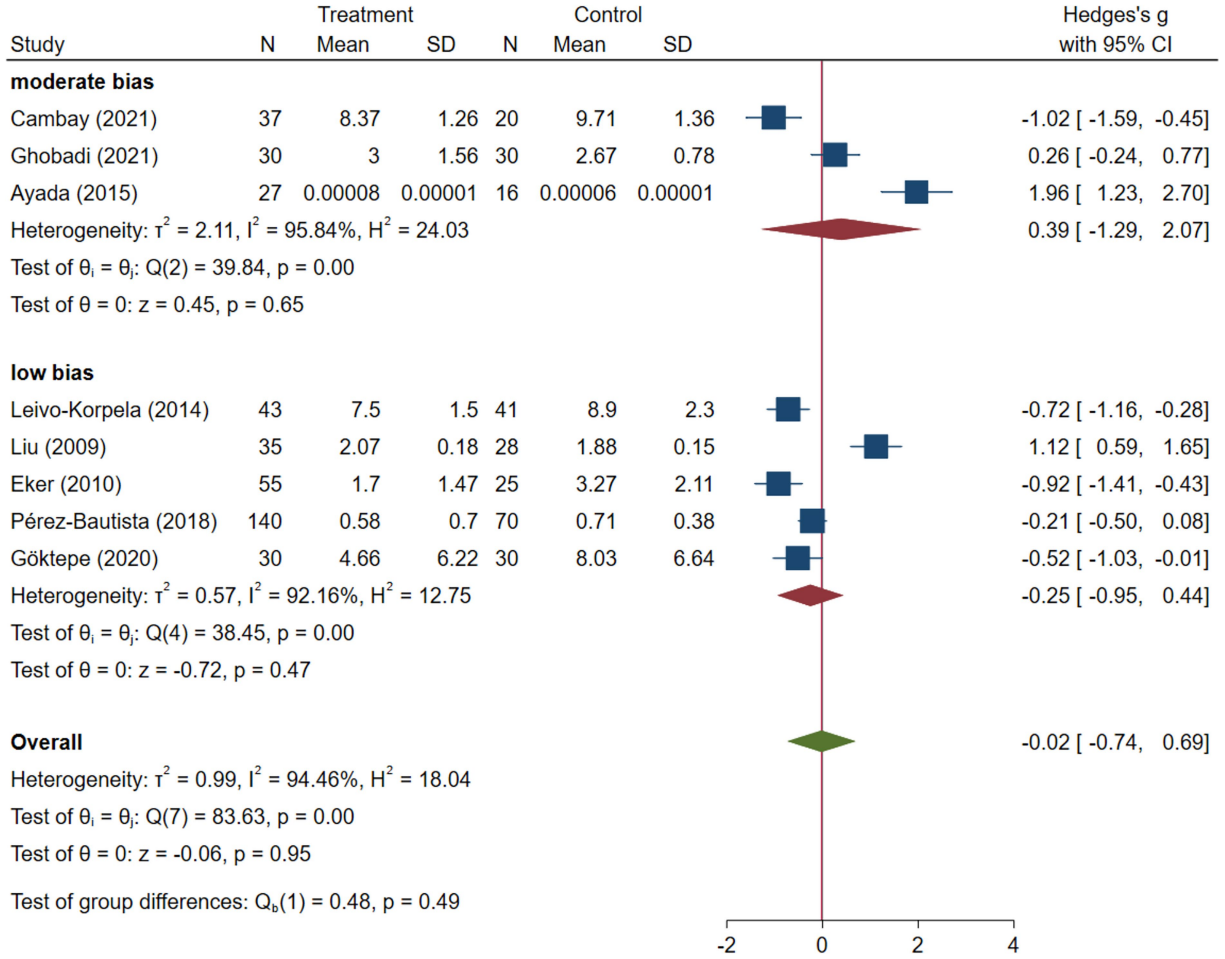
Subgroup analysis of COPD studies based on risk of bias.

**Figure 8:**
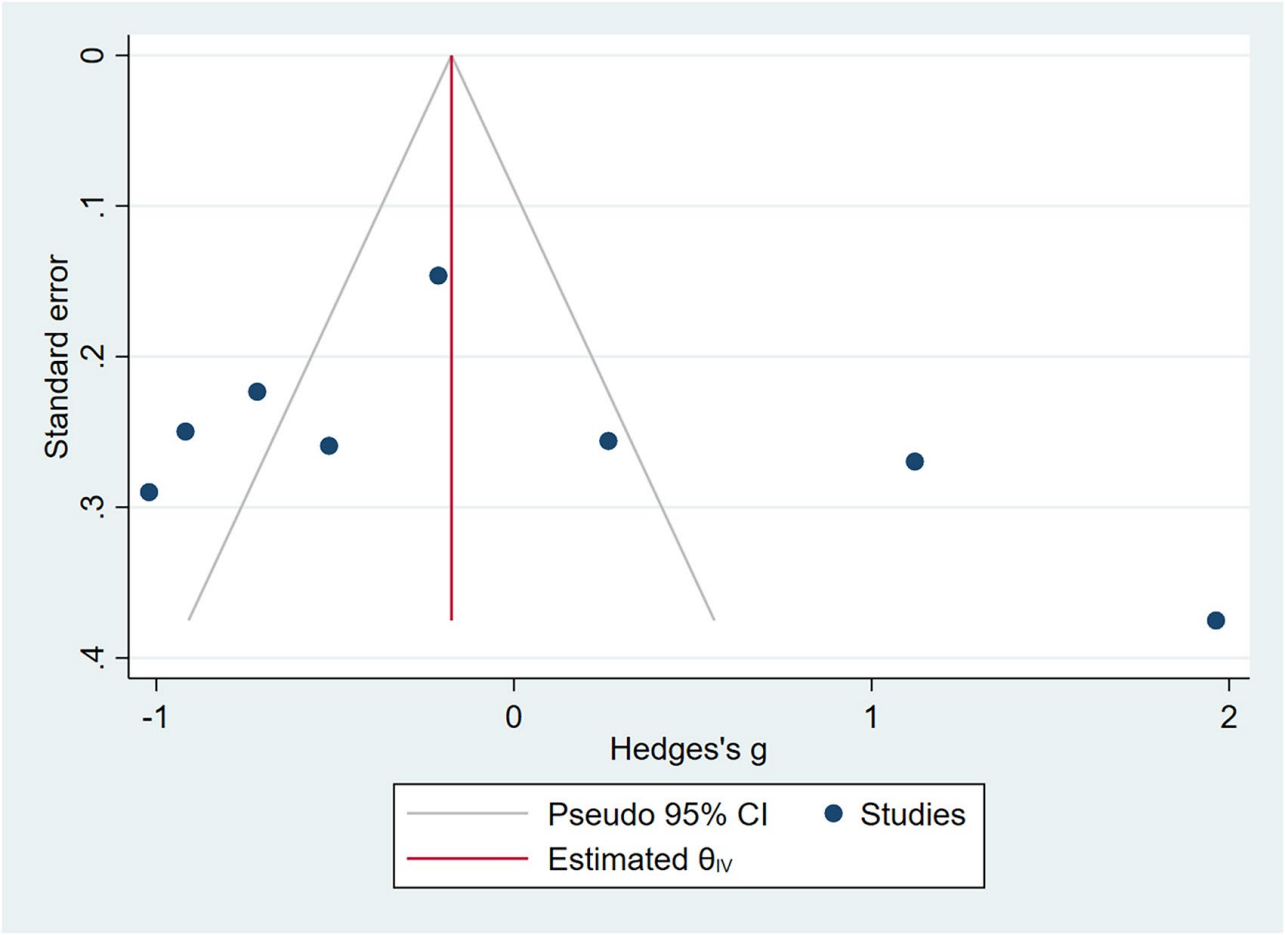
Funnel plot of COPD studies.

COPD analysis included 397 cases and 260 healthy controls. Regarding the population in COPD studies, all studies were within a close range of population, except Pérez-Bautista’s (28) study, which had 140 COPD patients and 70 healthy controls. Considering age, all the study populations were of approximately the same age range, except Ayada’s (33), which did not report their population age. All the studies used ELISA to assess visfatin levels, except two studies, which reported immunoassay (28) and enzyme immunoassay (8) as their assessment method. The random-effects analysis of COPD studies, which is depicted in Figure 2, showed no significant difference between the COPD group and healthy controls regarding serum visfatin levels (effect size = −0.02, %95 CI: [−0.74, 0.69], *p* = 0.95). Figure 3 represents the random-effects subgroup analysis of COPD studies based on study methodology. Neither case control (effect size = −0.37, %95 CI: [−1.63, 0.89], *p* = 0.56) nor cross-sectional (effect size = 0.10, %95 CI: [−0.80, 1.00], *p* = 0.83) showed any significant difference between patients’ and healthy controls’ visfatin levels. Also, no difference was observed between cross-sectional and case–control studies (*p* = 0.55). Figure 4 presents the leave-one-out sensitivity analysis of COPD studies. The analysis demonstrated that the overall findings of the meta-analysis were robust. Excluding any single study did not significantly affect the overall effect size or its statistical significance. Figure 5 is the forest plot of random-effects subgroup analysis on COPD studies that only involved male participants, which showed no significant statistical difference (effect size = −0.09, %95 CI: [−1.04, 0.86], *p* = 0.85). A random-effects subgroup analysis on COPD studies based on mean BMI is shown in Figure 6. Being overweight was defined as a mean BMI of participants > 24.9. In case the study did not report the mean BMI of all participants together, we used Cochrane’s formula to combine the means of patients and controls (34). This subgroup meta-analysis showed no difference in terms of serum visfatin between patients and healthy controls in normal weight (effect size = −0.27, %95 CI: [−1.64, 1.09], *p* = 0.70) or overweight (effect size = −0.30, %95 CI: [−0.69, 0.10], *p* = 0.14) studies. Random-effects subgroup analysis on COPD studies based on the risk of bias is depicted in Figure 7. Neither moderate risk (effect size = −0.25, %95 CI: [−0.95, 0.44], *p* = 0.47) nor high risk (effect size = 0.39, %95 CI: [−1.29, 2.07], *p* = 0.65) studies showed a significant difference between patients’ and controls’ visfatin levels. Meta regression of COPD studies for covariates, mean BMI of participants, male ratio, and mean age of participants, is shown in Table 2. For mean BMI, a coefficient of −0.106 (SE = 0.120, *p* = 0.120) was observed, indicating no statistically significant relationship with serum visfatin levels. The male ratio showed a coefficient of −0.014 (SE = 0.995, *p* = 0.989) with no significant association. Similarly, mean age had a coefficient of 0.054 (SE = 0.062, *p* = 0.381) with no significant effect on serum visfatin levels. The funnel plot of COPD studies illustrated heterogeneity in the results (Figure 8). Egger’s test provided evidence of small-study effects or publication bias in this meta-analysis (*p* = 0.0142).

**Table 2:** Meta regression of COPD studies for covariates mean BMI of participants, male ratio, and mean age of participants.

Covariate	Number of studies	Coefficient ± SE [95% CI]	*p*-value
Mean BMI of participants	7 of 8	−0.106 ± 0.120 [−0.342, 0.130]	0.379
Male ratio	6 of 8	−0.014 ± 0.995 [−1.936, 1.964]	0.989
Mean age of participants	6 of 8	0.054 ± 0.062 [−0.067, 0.176]	0.381

No covariates showed a significant effect on studies results.

### 3.3 Asthma

**Figure 9:**
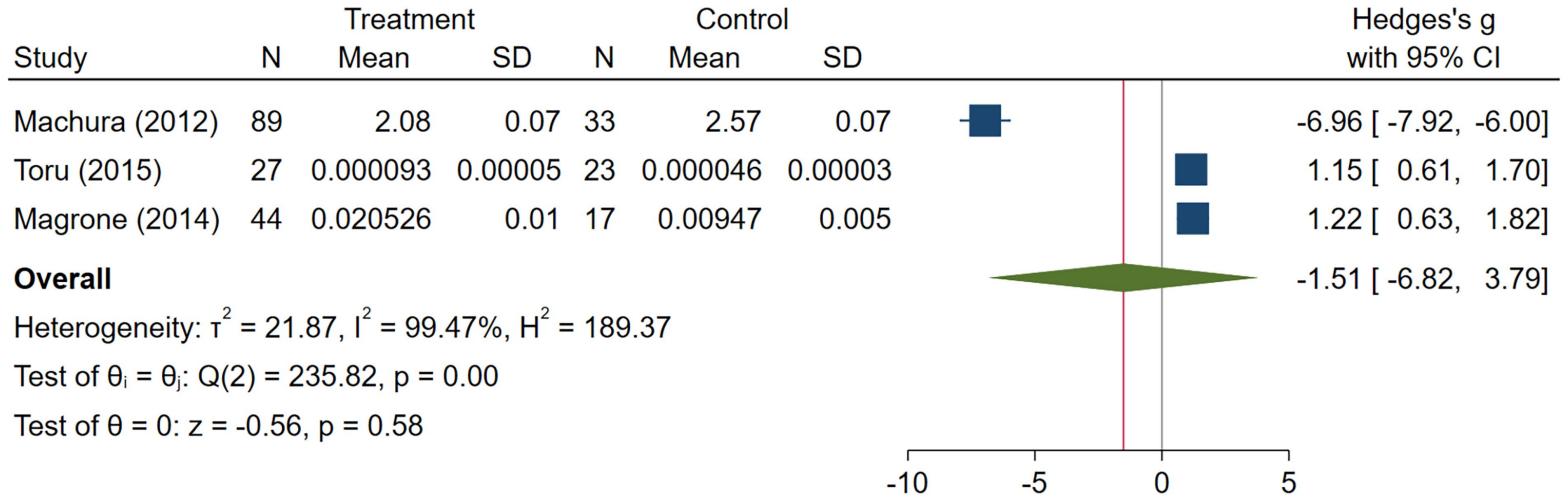
Random effects meta-analysis of asthma studies.

**Figure 10:**
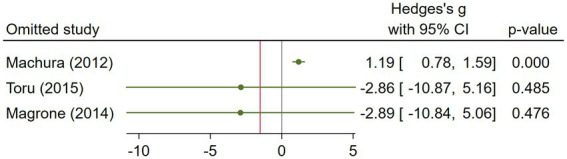
Leave-one-out sensitivity analysis of asthma studies.

**Figure 11:**
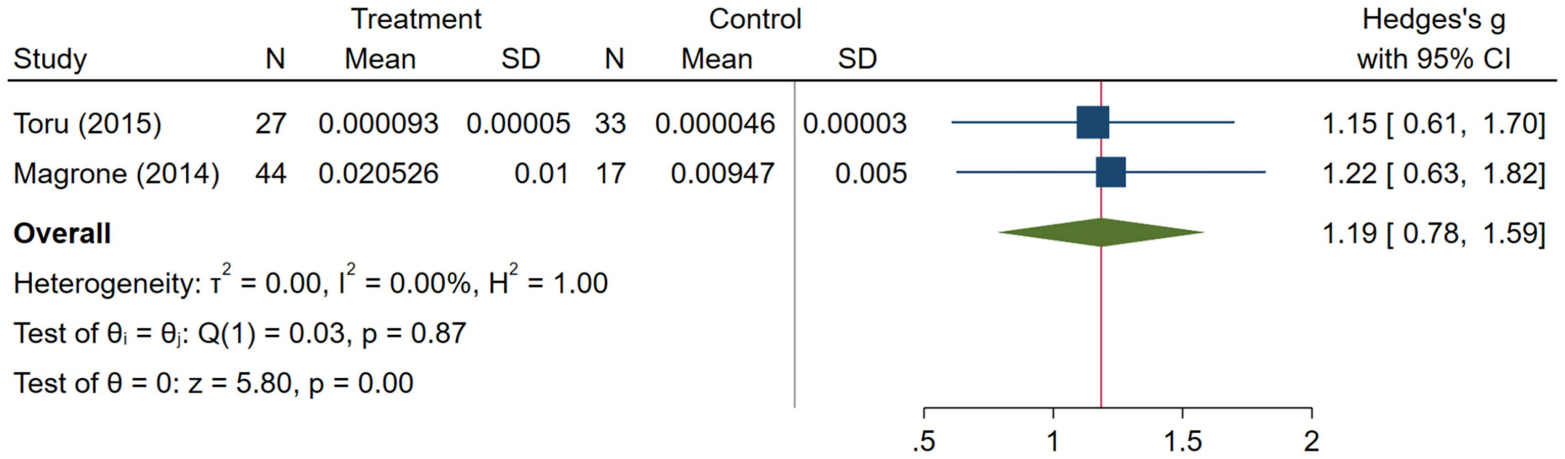
Random effects meta-analysis of asthma studies after excluding Machura et al. (23).

The asthma subgroup analysis comprised 160 cases and 73 healthy individual controls. One study included children (23), one included middle-aged participants (17), and one did not report the age (32). Magrone et al. (32) did not report the method they used to assess serum visfatin levels, and the rest used ELISA. Regarding the study population, Machura et al. (23) had a larger sample size (n = 122), which was almost twofold of the two other studies. A random-effects analysis of asthma studies showed no significant difference between the asthma group and healthy controls in terms of serum visfatin levels (effect size = −1.51, %95 CI: [−6.82, 3.79], *p* = 0.58) (Figure 9). The sensitivity analysis of asthma studies by leave-one-out method showed that when the study by Machura et al. was excluded, the serum level of visfatin was higher in asthma patients than healthy controls (effect size = 1.19, %95 CI: [0.78, 1.59], *p* < 0.01) (Figures 10, 11). We did not provide any leave-one-out analysis or forest plots for asthma due to the limited number of studies.

### 3.4 Pneumonia

**Figure 12:**
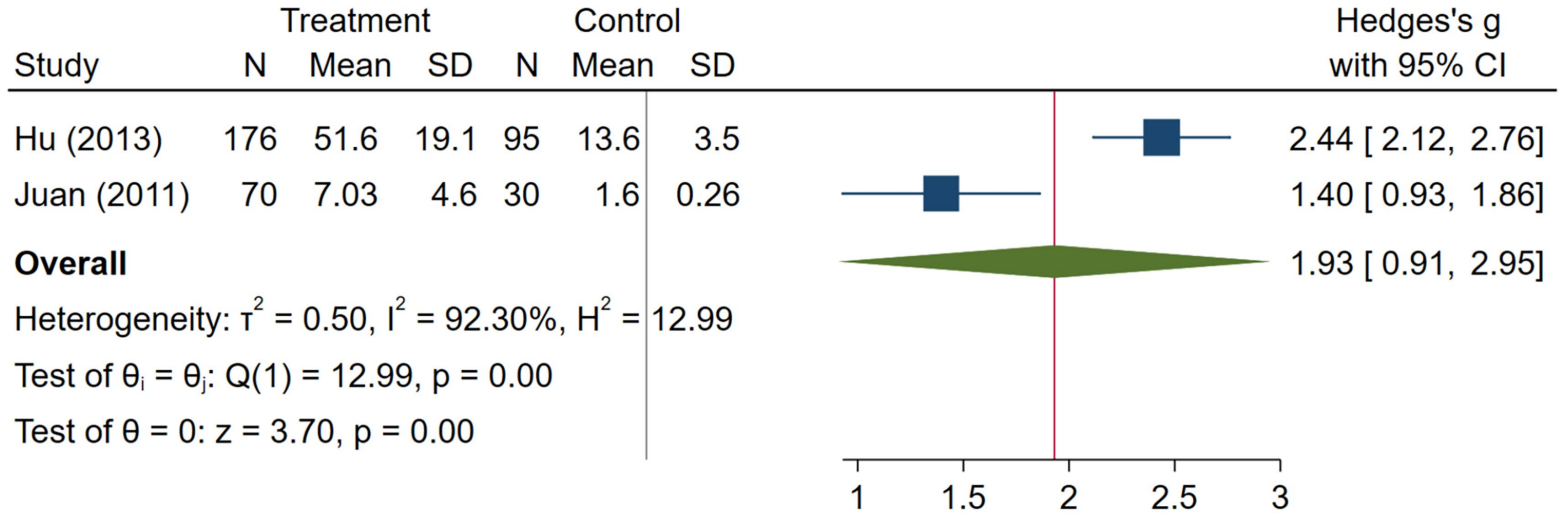
Random effects meta-analysis of pneumonia studies.

Pneumonia subgroup analysis included 246 cases and 125 healthy controls. Hu et al. (18) used older participants as their patients, but did not report the age of their healthy controls. Juan et al. (31) included middle-aged participants in their study. Hu et al. (18) et al. reported enzyme immunoassay as their method to measure serum visfatin levels, while the other study reported ELISA (31). Serum visfatin levels were significantly higher in pneumonia patients compared to healthy controls (effect size = 1.93, %95 CI: [0.91, 2.95], *p* < 0.01) (Figure 12). We did not provide any leave-one-out analysis or forest plots for pneumonia due to the limited number of studies.

## 4 Discussion

Our subgroup analysis for COPD was heterogeneous and yielded inconclusive results. Similarly, asthma subgroup analysis did not reveal any significant difference in serum visfatin between asthma patients and healthy controls. However, subgroup analysis of pneumonia demonstrated a significant increase in serum visfatin levels among pneumonia patients.

### 4.1 COPD

Our meta-analysis on the levels of visfatin in COPD showed heterogeneity, without a definitive result. Three studies showed significantly increased visfatin levels in COPD patients compared to healthy controls (15, 30, 33), one was non-significant (28), and four exhibited significantly reduced visfatin levels (8, 22, 27, 29). It appears that differences in study populations, methods, and visfatin measurement techniques caused these discrepancies. Future studies using the same methodology should control for confounding factors such as underlying diseases or medications that may alter adipokine levels (35).

It is suggested that increasing COPD severity is associated with a significant rise in IL-6 and visfatin. Also, serum visfatin levels show a negative association with SpO2 and FEV1 (30), while showing an association with IL-6 (8, 30), TNF-*α* (8, 15), IL-8 (8), and CRP (15). Decreased SpO2 may be one of the explanations for increased visfatin levels, since evidence suggests that elevated visfatin levels are induced by hypoxia-inducible factor-1 (HIF-1) (36). Additionally, inflammatory cytokines like TNF-α, IL-6, IL-1β, and lipopolysaccharide can stimulate visfatin production (8, 15). Moreover, visfatin inhibits neutrophil apoptosis, potentially contributing to inflammation in COPD patients (8). Ghobadi’s study reveals that during COPD exacerbation, visfatin levels rise in stages III–IV COPD GOLD criteria, with a significant association with IL-6. This suggests a pivotal role of visfatin in perpetuating and advancing inflammation in COPD. Elevated visfatin levels may result from systemic or local inflammation in COPD patients (30). However, we observed no statistically significant difference between the COPD group and the healthy controls regarding serum visfatin levels, and visfatin cannot be used as a biomarker for the diagnosis of COPD according to the available findings.

Visfatin shows varying circulating levels between obese and normal-weight individuals (37–39). In the study by Leivo-Korpela et al., it was observed that plasma visfatin levels exhibited a reduction in slightly overweight males diagnosed with COPD when compared to healthy controls who had similar BMI profiles (8). Liu et al. and Eker et al. reported consistent findings, indicating significantly lower visfatin levels in normal-weight or slightly overweight men with COPD. Conversely, underweight men diagnosed with COPD showed higher visfatin levels (15, 27). However, Leivo-Korpela et al. and Liu et al. showed that there was no association between visfatin and BMI in COPD patients (8, 15). The higher visfatin in underweight COPD patients reported by Liu et al. may be explained by the association of lower BMI with more severe systemic inflammation in COPD (15, 40). Our subgroup meta-analysis on normal weight and overweight patients and meta-regression on the mean BMI of participants did not show a strong association between BMI and visfatin levels.

### 4.2 Asthma

Our meta-analysis showed that visfatin levels do not differ between asthma patients and healthy subjects. Due to the limited number of studies, varying populations in terms of age, gender, and BMI, different methodologies, visfatin measurement techniques, and the failure to account for confounding factors, it is impossible to draw definitive conclusions on this topic.

According to the available findings, inflammation plays a decisive role in the development of asthma. Various immune cells, including CD8 + T, Th1, Th17, and Th2 cells, as well as non-classical lymphocytes like natural killer T (NKT) cells, contribute to the inflammatory cascade. Th2 cells are traditionally associated with asthma pathogenesis, promoting airway inflammation and hyperresponsiveness, while Th1 cells exert regulatory effects. The emergence of Th17 cells adds complexity, with their role in asthma still being elucidated. Furthermore, non-classical lymphocytes like NKT cells contribute to airway inflammation and hyperresponsiveness, particularly in response to allergens (41). On the other hand, visfatin plays a significant role in inflammation (42). Moschen et al. found that visfatin induces the expression of pro-inflammatory cytokines like IL-6, TNF-*α*, and IL-1β, and also anti-inflammatory cytokines such as IL-1 and IL-10 receptor antagonist in monocytes. Furthermore, visfatin stimulates the upregulation of co-stimulatory molecules pivotal for T cell activation and acts as a chemoattractant for monocytes and B cells. Experiments in mice showed that administering recombinant murine visfatin led to higher circulating IL-6 levels and increased IL-6 mRNA expression in the small intestine (4). Visfatin synthesized by neutrophils in response to inflammatory stimuli inhibits apoptosis triggered by various inflammatory agents and extends the lifespan of neutrophils, as seen in septic critically ill patients (13). Visfatin also plays a role in asthma-induced airway remodeling by enhancing fibroblast and smooth muscle cell activity, which may increase airway wall thickening (13, 43).

Our sensitivity analysis showed that after excluding Machura et al.’s study (23), the visfatin levels were significantly higher in asthmatic patients compared to healthy controls. Machura’s study (23) showed a decreased level of visfatin levels, in contrast to Toru’s study (17), which may be due to BMI differences that were higher in both cases and controls of Toru’s study. Research shows a direct correlation between BMI and asthma, suggesting that higher body weight increases the risk of asthma (44). Furthermore, Schachter et al. discovered that asthma symptoms worsened as BMI levels increased (45). Magrone et al. observed that serum visfatin levels increased as the BMI values of asthmatic children increased (32). Moreover, Samareh Fekri et al. showed that as BMI scores increased from underweight to overweight and obese categories, plasma visfatin concentrations showed an upward trend. However, this rise did not reach statistical significance (7).

### 4.3 Pneumonia

Our meta-analysis demonstrated a significant increase in serum visfatin levels among pneumonia patients compared to healthy controls.

Hu et al. showed that significantly elevated admission plasma visfatin levels were observed in community-acquired pneumonia patients compared to healthy controls. Admission plasma visfatin levels were reliable and independent predictors for identifying patients at risk of 30-day mortality. Visfatin’s predictive ability was comparable to that of the PSI and APACHE II scores, indicating its potential as a new prognostic biomarker (18). Juan et al. demonstrated that visfatin levels in plasma were higher in severe pneumonia compared to non-severe pneumonia (31). One explanation is that ventilation/perfusion imbalance, which causes hypoxia, is common in patients with severe pneumonia. Additionally, visfatin gene activation by HIF-1 under hypoxic conditions contributes to increased visfatin expression (4). Severe pneumonia, often complicated by infection and hypoxia, activates inflammatory cells, which trigger systemic inflammatory response syndrome (SIRS). Delayed PMN neutrophil apoptosis can lead to a harmful respiratory burst, intensifying SIRS (46). Visfatin inhibits neutrophil apoptosis by decreasing caspases-8 and caspases-3 activities. Moreover, visfatin prolongs neutrophil survival, activates them, increases intercellular adhesion molecule expression, and enhances PMN adhesion to the vascular endothelium (46, 47). Juan’s study showed higher levels of both visfatin and neutrophils in patients with severe pneumonia, with a positive correlation. This implies that visfatin might contribute to the inflammatory response by enhancing neutrophil function. Moreover, they showed associations between plasma visfatin levels, CRP, and APACHE II score, suggesting a prognostic role and a biomarker for assessing disease severity (31).

### 4.4 Limitations and strengths

Our study investigated the role of a novel inflammatory biomarker in several pulmonary diseases and performed subgroup analysis and meta-regression on proper covariates. However, the study had several limitations. First, due to the small number of qualified studies, particularly on asthma and pneumonia, it was not possible to perform proper subgroup analyses based on treatment response or disease severity. Second, among COPD studies, while six studies had visfatin levels in almost the same range (8, 15, 22, 27, 29, 30), Ayada’s (33) study reported that visfatin levels were much lower than those of other studies. It seems that different materials and methods could cause the differences in the results. Another limitation is that we did not search CENTRAL or Embase. However, since all included studies happened to be observational, and our topic was not intervention-focused, we believe it is unlikely that omission of these databases led to missing eligible studies. We recommend conducting further research to evaluate visfatin levels in acute conditions, including acute exacerbation of COPD, acute exacerbation of asthma, severe pneumonia, and ARDS.

## 5 Conclusion

Circulating levels of visfatin may not be associated with COPD and asthma, but may be associated with pneumonia. However, there are still few studies on the levels of visfatin in COPD, asthma, and pneumonia patients, and there is a need for further investigation.

## Data Availability

The original contributions presented in the study are included in the article/Supplementary material, further inquiries can be directed to the corresponding author/s.
