# Supplementary material for: Visfatin levels in pulmonary disease: a systematic review and meta-analysis
Source: Front Med (Lausanne). 2025 Sep 19;12:1541595. doi: 10.3389/fmed.2025.1541595 (PMC12491028; doi:10.3389/fmed.2025.1541595)
Supplement: Supplementary file 1 [file Table_1.DOCX]

| **Name of Database** | | |
| --- | --- | --- |
| **# of results** | **Query** | **Search** |
|  | “Nicotinamide Phosphoribosyltransferase” OR “Pre-B-Cell Colony-Enhancing Factor” OR “Pre B Cell Colony Enhancing Factor” OR “Visfatin” OR “NAmPRTase” OR “NMN Pyrophosphorylase” OR “NAMPT” OR “PBEF1” OR “eNAMPT” OR “iNAMPT” OR “1110035O14Rik” OR “PBEF” OR “VF” OR “EC 2.4.2.12” OR “EC-2.4.2.12” OR “2.4.2.12” OR “NAMPT_HUMAN” OR “P43490” | **#1** (visfatin) |
|  | “asthma” | **#2** (asthma) |
|  | “Chronic Obstructive Lung Disease” OR “Chronic Obstructive Pulmonary Disease” OR “COAD” OR “COPD” OR “Chronic Obstructive Airway Disease” OR “Chronic Airflow Obstruction” OR “Asthma-COPD Overlap Syndrome” OR “Asthma Chronic Obstructive Pulmonary Disease Overlap Syndrome” OR “Asthma-Chronic Obstructive Pulmonary Disease Overlap Syndrome” OR “Asthma COPD Overlap Syndrome” OR “emphysema” OR “chronic airflow limitation” OR “ACOS” OR “bronchitis” OR “CB” | **#3** (chronic obstructive pulmonary disease) |
|  | “Bronchiectasis” OR “Bronchiectases” | **#4** (bronchiectasis) |
|  | “pneumonia” OR “Pneumonitis” OR “Pneumonitides” OR “Pulmonary Inflammation” OR “Lung Inflammation” OR “Respiratory Tract Infections” | **#5** (pneumonia) |
|  | “Interstitial lung disease” OR “Diffuse Parenchymal Lung Disease” OR “ILD” | **#6** (interstitial lung disease) |
|  | **#1 AND (#2 OR #3 OR #4 OR #5 OR #6)** | **Final** |

| **PubMed** | | | | |
| --- | --- | --- | --- | --- |
| **# of results** | | **Query** | | **Search** |
| 16,872 | | "Nicotinamide Phosphoribosyltransferase"[Mesh] OR "Nicotinamide Phosphoribosyltransferase"[Title/Abstract] OR "Pre-B-Cell Colony-Enhancing Factor"[Title/Abstract] OR "Pre B Cell Colony Enhancing Factor"[Title/Abstract] OR "Visfatin"[Title/Abstract] OR "NAmPRTase"[Title/Abstract] OR "NMN Pyrophosphorylase"[Title/Abstract] OR "NAMPT"[Title/Abstract] OR "PBEF1"[Title/Abstract] OR "eNAMPT"[Title/Abstract] OR "iNAMPT"[Title/Abstract] OR "1110035O14Rik"[Title/Abstract] OR "PBEF"[Title/Abstract] OR "VF"[Title/Abstract] OR "EC 2.4.2.12"[Title/Abstract] OR "EC-2.4.2.12"[Title/Abstract] OR "2.4.2.12"[Title/Abstract] OR "NAMPT_HUMAN"[Title/Abstract] OR "P43490"[Title/Abstract] | | **#1** (visfatin) |
| 198,108 | | "Asthma"[Mesh] OR "asthma"[Title/Abstract] | | **#2** (asthma) |
| 161,317 | | "Pulmonary Disease, Chronic Obstructive"[Mesh] OR "Chronic Obstructive Lung Disease"[Title/Abstract] OR "Chronic Obstructive Pulmonary Disease"[Title/Abstract] OR "COAD"[Title/Abstract] OR "COPD"[Title/Abstract] OR "Chronic Obstructive Airway Disease"[Title/Abstract] OR "Chronic Airflow Obstruction"[Title/Abstract] OR "Asthma-COPD Overlap Syndrome"[Title/Abstract] OR "Asthma Chronic Obstructive Pulmonary Disease Overlap Syndrome"[Title/Abstract] OR "Asthma-Chronic Obstructive Pulmonary Disease Overlap Syndrome"[Title/Abstract] OR "Asthma COPD Overlap Syndrome"[Title/Abstract] OR "emphysema"[Title/Abstract] OR "chronic airflow limitation"[Title/Abstract] OR "ACOS"[Title/Abstract] OR "bronchitis"[Title/Abstract] OR "CB"[Title/Abstract] | | **#3** (chronic obstructive pulmonary disease) |
| 15,779 | | "Bronchiectasis"[Mesh] OR "Bronchiectasis"[Title/Abstract] OR "Bronchiectases"[Title/Abstract] | | **#4** (bronchiectasis) |
| 445,862 | | "Pneumonia"[Mesh] OR "pneumonia"[Title/Abstract] OR "Pneumonitis"[Title/Abstract] OR "Pneumonitides"[Title/Abstract] OR "Pulmonary Inflammation"[Title/Abstract] OR "Lung Inflammation"[Title/Abstract] OR "Respiratory Tract Infections"[Title/Abstract] | | **#5** (pneumonia) |
| 91,136 | | "Lung Diseases, Interstitial"[Mesh] OR "Interstitial lung disease"[Title/Abstract] OR "Diffuse Parenchymal Lung Disease"[Title/Abstract] OR "ILD"[Title/Abstract] | | **#6** (interstitial lung disease) |
| **194** | | **#1 AND (#2 OR #3 OR #4 OR #5 OR #6)** | | **Final** |
| **Web of Science** | | | | |
| **# of results** | **Query** | | **Search** | |
| 17,709 | TI=(“Nicotinamide Phosphoribosyltransferase” OR “Pre-B-Cell Colony-Enhancing Factor” OR “Pre B Cell Colony Enhancing Factor” OR “Visfatin” OR “NAmPRTase” OR “NMN Pyrophosphorylase” OR “NAMPT” OR “PBEF1” OR “eNAMPT” OR “iNAMPT” OR “1110035O14Rik” OR “PBEF” OR “VF” OR “EC 2.4.2.12” OR “EC-2.4.2.12” OR “2.4.2.12” OR “NAMPT_HUMAN” OR “P43490”) OR AB=(“Nicotinamide Phosphoribosyltransferase” OR “Pre-B-Cell Colony-Enhancing Factor” OR “Pre B Cell Colony Enhancing Factor” OR “Visfatin” OR “NAmPRTase” OR “NMN Pyrophosphorylase” OR “NAMPT” OR “PBEF1” OR “eNAMPT” OR “iNAMPT” OR “1110035O14Rik” OR “PBEF” OR “VF” OR “EC 2.4.2.12” OR “EC-2.4.2.12” OR “2.4.2.12” OR “NAMPT_HUMAN” OR “P43490”) | | **#1** (visfatin) | |
| 170,120 | TI=(“asthma”) OR AB=(“asthma”) | | **#2** (asthma) | |
| 159,032 | TI=(“Chronic Obstructive Lung Disease” OR “Chronic Obstructive Pulmonary Disease” OR “COAD” OR “COPD” OR “Chronic Obstructive Airway Disease” OR “Chronic Airflow Obstruction” OR “Asthma-COPD Overlap Syndrome” OR “Asthma Chronic Obstructive Pulmonary Disease Overlap Syndrome” OR “Asthma-Chronic Obstructive Pulmonary Disease Overlap Syndrome” OR “Asthma COPD Overlap Syndrome” OR “emphysema” OR “chronic airflow limitation” OR “ACOS” OR “bronchitis” OR “CB”) OR AB=(“Chronic Obstructive Lung Disease” OR “Chronic Obstructive Pulmonary Disease” OR “COAD” OR “COPD” OR “Chronic Obstructive Airway Disease” OR “Chronic Airflow Obstruction” OR “Asthma-COPD Overlap Syndrome” OR “Asthma Chronic Obstructive Pulmonary Disease Overlap Syndrome” OR “Asthma-Chronic Obstructive Pulmonary Disease Overlap Syndrome” OR “Asthma COPD Overlap Syndrome” OR “emphysema” OR “chronic airflow limitation” OR “ACOS” OR “bronchitis” OR “CB”) | | **#3** (chronic obstructive pulmonary disease) | |
| 9,490 | TI=(“Bronchiectasis” OR “Bronchiectases”) OR AB=(“Bronchiectasis” OR “Bronchiectases”) | | **#4** (bronchiectasis) | |
| 164,853 | TI=(“pneumonia” OR “Pneumonitis” OR “Pneumonitides” OR “Pulmonary Inflammation” OR “Lung Inflammation” OR “Respiratory Tract Infections”) OR AB=(“pneumonia” OR “Pneumonitis” OR “Pneumonitides” OR “Pulmonary Inflammation” OR “Lung Inflammation” OR “Respiratory Tract Infections”) | | **#5** (pneumonia) | |
| 17,042 | TI=(“Interstitial lung disease” OR “Diffuse Parenchymal Lung Disease” OR “ILD”) OR AB=(“Interstitial lung disease” OR “Diffuse Parenchymal Lung Disease” OR “ILD”) | | **#6** (interstitial lung disease) | |
| **127** | **#1 AND (#2 OR #3 OR #4 OR #5 OR #6)** | | **Final** | |
| **Scopus** | | | | |
| **# of results** | **Query** | | **Search** | |
| 28,038 | TITLE-ABS-KEY ( "Nicotinamide Phosphoribosyltransferase" OR "Pre-B-Cell Colony-Enhancing Factor" OR "Pre B Cell Colony Enhancing Factor" OR "Visfatin" OR "NAmPRTase" OR "NMN Pyrophosphorylase" OR "NAMPT" OR "PBEF1" OR "eNAMPT" OR "iNAMPT" OR "1110035O14Rik" OR "PBEF" OR "VF" OR "EC 2.4.2.12" OR "EC-2.4.2.12" OR "2.4.2.12" OR "NAMPT_HUMAN" OR "P43490" ) | | **#1** (visfatin) | |
| 295,992 | TITLE-ABS-KEY ( "asthma" ) | | **#2** (asthma) | |
| 300,037 | TITLE-ABS-KEY ( "Chronic Obstructive Lung Disease" OR "Chronic Obstructive Pulmonary Disease" OR "COAD" OR "COPD" OR "Chronic Obstructive Airway Disease" OR "Chronic Airflow Obstruction" OR "Asthma-COPD Overlap Syndrome" OR "Asthma Chronic Obstructive Pulmonary Disease Overlap Syndrome" OR "Asthma-Chronic Obstructive Pulmonary Disease Overlap Syndrome" OR "Asthma COPD Overlap Syndrome" OR "emphysema" OR "chronic airflow limitation" OR "ACOS" OR "bronchitis" OR "CB" ) | | **#3** (chronic obstructive pulmonary disease) | |
| 25,276 | TITLE-ABS-KEY ( "Bronchiectasis" OR "Bronchiectases" ) | | **#4** (bronchiectasis) | |
| 604,521 | TITLE-ABS-KEY ( "pneumonia" OR "Pneumonitis" OR "Pneumonitides" OR "Pulmonary Inflammation" OR "Lung Inflammation" OR "Respiratory Tract Infections" ) | | **#5** (pneumonia) | |
| 31,720 | TITLE-ABS-KEY ( "Interstitial lung disease" OR "Diffuse Parenchymal Lung Disease" OR "ILD" ) | | **#6** (interstitial lung disease) | |
| **331** | **#1 AND (#2 OR #3 OR #4 OR #5 OR #6)** | | **Final** | |

Table S1 – detailed search strategy
