# Supplementary material for: Visfatin levels in pulmonary disease: a systematic review and meta-analysis
Source: Front Med (Lausanne). 2025 Sep 19;12:1541595. doi: 10.3389/fmed.2025.1541595 (PMC12491028; doi:10.3389/fmed.2025.1541595)
Supplement: Supplementary file 2 [file Table_2.DOCX]

| **Cross-sectioanl studies** | | | | | | | | | |
| --- | --- | --- | --- | --- | --- | --- | --- | --- | --- |
| **STUDY** | **SELECTION (max 5 points)** | | | | **COMPARABILITY (max 1 points)** | **OUTCOME (max 3 points)** | | | **SCORE (out of 9)** |
|  | Representativeness | Sample size | Non-response rate | Ascertainment of the screening/surveillance tool | The potential confounders were investigated by subgroup analysis or multivariable analysis. | Assessment of outcome | Statistical test | |  |
| Leivo-Korpela (2014) (1) | 1 | 0 | 0 | 2 | 1 | 2 | 1 | | 7 |
| Liu (2009) (2) | 1 | 0 | 0 | 2 | 1 | 2 | 1 | | 7 |
| Eker (2010) (3) | 1 | 0 | 0 | 2 | 1 | 2 | 1 | | 7 |
| Machura (2012) (4) | 1 | 1 | 1 | 2 | 1 | 2 | 1 | | 9 |
| Toru (2015) (5) | 0 | 0 | 0 | 2 | 0 | 2 | 1 | | 5 |
| Pérez-Bautista (2018) (6) | 0 | 1 | 0 | 2 | 1 | 2 | 1 | | 7 |
| Göktepe (2020) (7) | 1 | 0 | 0 | 2 | 1 | 2 | 1 | | 7 |
| Juan (2011) (8) | 1 | 1 | 0 | 2 | 0 | 2 | 1 | | 7 |
| Magrone (2014) (9) | 1 | 0 | 0 | 2 | 0 | 2 | 1 | | 6 |
| Ayada (2015) (10) | 1 | 0 | 0 | 2 | 0 | 2 | 1 | | 6 |
| Vantaggiato (2023) (11) | 1 | 0 | 0 | 2 | 0 | 2 | 1 | | 6 |
| **Case-control studies** | | | | | | | | | |
| **STUDY** | **SELECTION (max 4 points)** | | | | **COMPARABILITY (max 2 points)** | **OUTCOME (max 3 points)** | | | **SCORE (out of 9)** |
|  | Adequate case definition | Representativeness | Selection of controls | Definition of controls | Comparability the basis of the design or analysis | Assessment of exposure | Same method of ascertainment for cases and controls | Non-Response rate |  |
| Cambay (2021) (12) | 1 | 1 | 0 | 1 | 2 | 0 | 1 | 0 | 6 |
| Hu (2013) (13) | 1 | 1 | 0 | 1 | 2 | 0 | 1 | 0 | 6 |
| Ghobadi (2021) (14) | 1 | 0 | 0 | 1 | 2 | 1 | 1 | 0 | 6 |

Table S2- quality assessment of included studies using Newcastle-Ottawa Scale

1. Leivo-Korpela S, Lehtimäki L, Hämälainen M, Vuolteenaho K, Kööbi L, Järvenpää R, et al. Adipokines NUCB2/nesfatin-1 and visfatin as novel inflammatory factors in chronic obstructive pulmonary disease. Mediators Inflamm. 2014;2014:232167.

2. Liu XJ, Ji YL, Chen J, Li SQ, Luo FM. Circulating visfatin in chronic obstructive pulmonary disease. Nutrition. 2009;25(4):373-8.

3. Eker S, Ayaz L, Tamer L, Ulubas B. Leptin, visfatin, insulin resistance, and body composition change in chronic obstructive pulmonary disease. Scand J Clin Lab Invest. 2010;70(1):40-4.

4. Machura E, Ziora K, Ziora D, Świtęochowska E, Halkiewicz F, Oświęcimska J, et al. Serum visfatin levels are decreased in schoolchildren with atopic asthma. Neuro Endocrinol Lett. 2012;33(5):559-64.

5. Toru U, Ayada C, Genc O, Sahin S, Arik O, Acat M, et al. Visfatin and ghrelin: can they be forthcoming biomarkers or new drug targets for asthma? Int J Clin Exp Med. 2015;8(4):6257-61.

6. Pérez-Bautista O, Montaño M, Pérez-Padilla R, Zúñiga-Ramos J, Camacho-Priego M, Barrientos-Gutiérrez T, et al. Women with COPD by biomass show different serum profile of adipokines, incretins, and peptide hormones than smokers. Respir Res. 2018;19(1):239.

7. Goktepe M, Korkmaz C, Zamani A, Demirbas S, Kilinc I. Evaluation of Serum Resistin, Visfatin, and Chemerin Levels in Patients with Lung Cancer and Chronic Obstructive Pulmonary Disease. Turk Thorac J. 2020;21(3):169-73.

8. Juan X, Lu YM, Shi JD, Deng XQ, Long W. Visfatin levels in patients with severe pneumonia. World J Emerg Med. 2011;2(2):132-6.

9. Magrone T, Simone M, Altamura M, Munno I. Characterization of the immune inflammatory profile in obese asthmatic children. Endocr Metab Immune Disord Drug Targets. 2014;14(3):187-95.

10. Ayada C, Toru U, Genc O, Sahin S, Arik O, Bulut I. Serum levels of leptin and visfatin in chronic obstructive pulmonary disease. Eur Resp J. 2015;46:2.

11. Vantaggiato L, Shaba E, Cameli P, Bergantini L, d’Alessandro M, Carleo A, et al. BAL Proteomic Signature of Lung Adenocarcinoma in IPF Patients and Its Transposition in Serum Samples for Less Invasive Diagnostic Procedures. Int J Mol Sci. 2023;24(2).

12. Cambay Z, Ilhan N, Susam S, Muz MH. BMI and adipocytokine changes in COPD exacerbation and stable COPD. Indian J Biochem Biophys. 2021;58(5):472-7.

13. Hu W, Liu CW, Su J, Lu J, Zhu Y, Liu BW. Elevated plasma visfatin concentrations in patients with community-acquired pneumonia. Peptides. 2013;43:8-12.

14. Ghobadi H, Mokhtari S, Aslani M. Serum levels of visfatin, sirtuin-1, and interleukin-6 in stable and acute exacerbation of chronic obstructive pulmonary disease. J Res Med Sci. 2021;26(1).
